# Editorial: The Impact of Immunosenescence and Senescence of Immune Cells on Responses to Infection and Vaccination

**DOI:** 10.3389/fragi.2022.882494

**Published:** 2022-05-12

**Authors:** Anshu Agrawal, Birgit Weinberger

**Affiliations:** ^1^ Division of Basic and Clinical Immunology, Department of Medicine, University of California, Irvine, Irvine, CA, United States; ^2^ Institute for Biomedical Aging Research, Universität Innsbruck, Innsbruck, Austria

**Keywords:** immunosenescence, T cell repertoire, antigen-specific T cells, vaccines, pneumococcal responses

Advancing age is characterized by changes in the innate and adaptive immune system termed immunosenescence, which contribute to the pathogenesis of various diseases, increased incidence of infections and reduced response to vaccinations. Antigen sensing, presentation and cytokine responses of innate cells are all altered with age leading to impaired responses to infection and vaccines. Aging affects individual T and B cells throughout their life-cycle, from alterations in hematopoiesis, maturation and homeostasis, to memory generation and effector functions as well as their interactions with other cell types, and the composition and repertoire of the adaptive immune cell compartments. Latent infection with Cytomegalovirus (CMV), has a profound impact on the aging immune system, particularly on the T cell compartment, but also other herpesviruses, e.g., Epstein-Barr virus (EBV) might play a role in T cell aging. Periodical reactivation of these viruses shapes the evolution of the T cell repertoire in aging leading to decreased diversity. The general dogma is that the T cell memory responses generated early in life remain stable as we age. Thus, one of the proposed strategies to improve vaccine responses in the elderly is to vaccinate early before these changes occur. However, Lanfermeijer et al. show that the T cell repertoire for CMV and EBV loses stability with age and becomes less diverse. They also demonstrate that CMV-infection is associated with a decreased diversity of EBV-specific CD8^+^ T cells highlighting the impact of CMV on the T cell response to other pathogens. In the past, life-long repeated antigenic stimulation (e.g., in the context of CMV-infection) was thought to be the only driver of terminal differentiation of T cells. These terminally differentiated T cells, which accumulate with aging, exhibit characteristics of senescence and are frequently thought to be dysfunctional. However, recent data show that senescent-like T cells acquire alternative functions, e.g. Natural Killer (NK) cell characteristics, which are independent of antigen specificity, and that antigen-independent bystander activation of T cells by cytokines may drive senescence as well as expression of NK-like activity in T cells (Abbas and Akbar). Replicative and cellular senescence is frequently studied in cell types such as fibroblasts, but over the last years hallmarks of aging such as DNA damage, the senescence-associated secretory phenotype (SASP), mitochondrial dysfunction, protein homeostasis etc., have also been investigated in immune cells and have been identified as important players in T cell aging. Using mice with a deficiency in the DNA excision-repair gene *Ercc1,* Pieren et al. show accumulation of Tregs with an aging-related phenotype and reduced T-cell responsiveness.

Immunosenescence contributes to increased incidence, morbidity and mortality of infections, e.g., of influenza, pneumococcal disease, herpes zoster and many more. He et al. investigate CD4^+^ T cells specific for the immunogenic pneumococcal protein AliB and show that proliferation of pneumococcus-specific T cells as well as cytokine production is impaired in older adults, while regulatory T cells, that suppress T cell activation and induction of protective cytokines like IFN-γ and IL-17 accumulate. Lambert et al. studied CD4^+^ T cell responses to pertussis in aging individuals. They also observed reduced frequencies of proliferating CD4^+^ T cells against *Bordetella pertussis,* but this was not due to an increase in T regulatory cells. Instead, there was a deficiency in the T cell activation markers suggesting that several mechanisms are impacting CD4^+^ T cell responses in the elderly. More importantly, they also showed that there is a gradual loss of memory against various pertussis epitopes decreasing the diversity of the pertussis repertoire similar to what was shown by Lanfermeijer et al. for EBV-specific CD8^+^ T cells. The two studies clearly highlight the importance of studying antigen-specific T cell repertoire responses in aging. An insight into the mechanisms underlying the selective loss of certain epitopes with age is essential to design effective vaccines against the right epitopes. We also need to understand whether this loss is selective to certain phenotypes of memory T cells. The contribution of innate immune cells such as dendritic cells (DCs) and macrophages in maintenance of the T cell memory pool is also an understudied area. The impact of age on infectious diseases recently received increased attention as old age is also a major risk factor for severe COVID-19 disease. Farheen et al. summarize the impact of an aged immune system on susceptibility to SARS-CoV-2 infection and the risk for complications. At the same time, many -but not all-vaccines show less immunogenicity and clinical efficacy/effectiveness in older adults. Antibody responses to influenza and pneumococcal vaccination are generally lower in older adults leading to decreased protection for this age group. Crooke et al. report that inflammasome activation in response to influenza vaccination is preserved in monocyte-derived macrophages from older adults and does not explain the lower antibody responses observed in this age group. They also found increased expression of certain inflammasome-related genes in females compared to males highlighting the need to study sex-related differences in immune responses. Development of vaccine adjuvants and delivery systems that are effective in the elderly is the need of the hour especially with increased longevity. Bhalla et al. suggest a novel formulation of Liposomal Encapsulation of Polysaccharides (LEPS) to improve immune responses to pneumococcal vaccine and demonstrate protective effects of LEPS against invasive and pulmonary pneumococcal infections in aged mice. It will be interesting to see whether this platform is effective in generation of respiratory immunity against other pathogens. Several vaccines are specifically recommended for older adults (e.g., against influenza, pneumococcal disease and herpes zoster). In addition, vaccines recommended for all adults, such as regular booster vaccinations against tetanus/diphtheria/pertussis or other diseases depending on the epidemiological/geographical situation are relevant for older adults. The number of older travelers is on the rise and senior travelers have specific risks linked to their age, overall health and travel patterns. However, data on efficacy/effectiveness of travel vaccines in older adults are scarce. Ecarnot et al. summarize the risk of major vaccine-preventable travel-associated infectious diseases and vaccines recommended for older travelers.

In summary, aging leads to the accumulation of dysfunctional T cells along with a reduction of antigen-specific T cell repertoire. The loss of naïve T cells is considered a major culprit. The intrinsic changes in T cells themselves as well as age-associated changes in antigen presenting cells (APCs) required to prime T cell responses are areas that need further studies to improve immune responses. In this regard, information regarding tissue-resident immunity is also lacking in aging. The changes in mucosal immune response, also needs further studying since it is becoming clear that the tissue microenvironment shapes the immune response and older adults are at high risk for respiratory infections. Changes in matrix components and accumulation of dysfunctional or senescent matrix cells such as fibroblasts as well as immune cells are increasingly being recognized for their role in various age-associated diseases. Novel strategies are needed to rejuvenate the aging immune system in order to prevent infections and other age-associated diseases and to ensure optimal protection by vaccination. One such strategy might be the use of senolytics that can selectively remove dysfunctional aged cells. Cancer therapies as well as drugs altering the metabolic state of cells to induce apoptosis are potential senolytics. However, development of technologies that can target senescent cells for delivery of senolytics will be essential for the success of this approach.

